# General practice recording of adverse childhood experiences: a retrospective cohort study of GP records

**DOI:** 10.3399/bjgpopen20X101011

**Published:** 2020-02-19

**Authors:** Andrea E Williamson, Ross McQueenie, David A Ellis, Alex McConnachie, Philip Wilson

**Affiliations:** 1 Senior Clinical University Lecturer, School of Medicine, Dentistry & Nursing, General Practice & Primary Care, University of Glasgow, Glasgow, UK; 2 GP and MO Addictions, NHS Greater Glasgow and Clyde, Glasgow, UK; 3 Research Associate, General Practice & Primary Care, University of Glasgow, Glasgow, UK; 4 Lecturer in Computational Social Science, Department of Psychology, Lancaster University, Lancaster, UK; 5 Professor of Clinical Trial Biostatistics, Robertson Centre for Biostatistics, University of Glasgow, Glasgow, UK; 6 Director of Centre for Rural Health, The Institute of Applied Health Sciences, University of Aberdeen, Aberdeen, UK

**Keywords:** General practice, adverse childhood experiences, appointments and schedules, healthcare utilization

## Abstract

**Background:**

Adverse childhood experiences (ACEs) are linked to negative health outcomes in adulthood. Poor engagement with services may, in part, mediate the association between adverse outcomes and ACEs. While appointment recording is comprehensive, it is not yet known if or how ACEs are recorded in the GP clinical record (GPR).

**Aim:**

To investigate recording of ACEs in the GPR and assess associations between available ACE-related Read codes and missed appointments.

**Design & setting:**

Retrospective cohort study of 824 374 anonymised GPRs. Nationally representative sample of 136 Scottish general practices; data collected 2013–2016.

**Method:**

Read codes were mapped onto ACE questionnaire and wider ACE-related domains. Natural language processing (NLP) was used to augment capture of non-Read-coded ACEs. Frequency counts and proportions of mapped codes, and associations of these with defined levels of missing GP appointments, are reported.

**Results:**

In total, 0.4% of patients had a record of any code that mapped onto the ACE questionnaire, contrasting with survey-reported rates of 47% in population samples. This increased only modestly by including inferred ACEs that related to safeguarding children concerns, wider aspects of ACEs, and adult consequences of ACEs. Augmentation via NLP did not substantially increase capture. Despite poor recording, there was an association between ever having an ACE code recorded and higher rates of missing GP appointments.

**Conclusion:**

General practices would require substantial support to implement the recording of ACEs in the GPR. This study adds to the evidence that patients who often miss appointments are more likely to be socially vulnerable.

## How this fits in

ACEs continue to generate significant research and policy attention due to links with social and health outcomes. This study shows that recording of ACEs is very sparse in Scottish GP clinical records. There is nevertheless an association between any recorded ACE and the likelihood of missing GP appointments, supporting previous evidence linking missed appointments with social vulnerability. Comprehensive recording of ACEs in future GPRs may be desirable but would require substantial resources.

## Introduction

Difficult experiences that threaten a person’s safety, especially if they occur over time and in childhood, are associated with negative life outcomes.^1^ A simple epidemiological measure has been used to correlate adversities experienced in childhood — ACEs — with health outcomes in adults. ACEs, quantified in the US among adult members of a Health Maintenance Organisation,^2^ and more recently at population level in several high income countries, including England^3^ and Wales,^4^ have proved to be an important predictor of poor physical and mental health. This evidence has encouraged public services to address ACEs by focusing on prevention, and mitigating the impact that ACEs have across the lifespan.^5,6^

ACEs are measured in adults using a questionnaire about experiences that occurred when they were aged <18 years. The standard form used in most settings is an 11-item questionnaire which covers the domains of childhood maltreatment (sexual, physical, and verbal abuse, and neglect), and exposure to care-giving adults at home who are struggling with serious concerns of their own (domestic violence, parental separation, substance use, mental health issues, and imprisonment).^4^ In the absence of questionnaire data, other sources may provide valuable insights. For example, in the UK NHS almost all people are registered with a general practice and their GPR follows them throughout life. The GPR contains information on each contact with a member of the practice team and details of care carried out elsewhere: diagnoses and life events are extracted from this information by practice personnel. Thus, the GPR is the most comprehensively coded health record available for individuals in the UK. No published research has examined ACE questionnaire information in the UK GPR.

The aim of this study was to report on the extraction of information from routine GPRs corresponding to ACE questionnaire domains, consequences of ACEs, and other factors relating to adversity in childhood. This was in the context of a study that investigated the epidemiology of missed GP appointments^7,8^ and associated health outcomes.^9^ The hypothesis was that the experience of adversity in childhood would be associated with high levels of missed GP appointments in adulthood, a measurable proxy of low engagement in care and potential mediator of adverse health outcomes. The conceptual framework of the ACE literature also underpins the theoretical perspective; that adverse experiences across the life course have a cumulative effect and can lead to ‘health harming behaviours’ such as poor engagement with services.

## Method

A total of 824 374 GPRs were extracted from a nationally representative sample of 136 general practices in Scotland over a 3-year period from September 2013 to September 2016. Data were extracted by a trusted third party (TTP) for the NHS, anonymised, and linked to a unique patient identifier in the Scottish NHS Safehaven for analysis. Details are documented in existing publications.^7–9^

### Read code extract

GPRs in the UK are currently coded using Read codes.^10^ Decisions about codes to include were initially made by one author and inclusion/exclusion decisions moderated by another. Both are GPs experienced in using Read codes and working with patients experiencing adversity.

The ACE questionnaire domains were mapped onto these codes: nine ACE domains mapped onto the 11 ACE questions (Table 1).^4^

**Table 1.** ACEs mapped to Read codes included in the study. All ACE questions were preceded by the statement ‘While you were growing up, before the age of 18 ...’

**ACE Questionnaire domains**	**Mapped Read codes**
**1.** **Sexual abuse**How often did anyone at least 5 years older than you (including adults) try to make you touch them sexually?How often did anyone at least 5 years older than you (including adults) force you to have any type of sexual intercourse (oral, anal, or vaginal)?How often did anyone at least 5 years older than you (including adults) ever touch you sexually? Once or more than once to any of the three questions	0AK3. Child prostituteZV4G4 [V]Problem related/alleged sex abuse13ZW. At risk of sexual abuse14 × 1. History of sexual abuse13WC. Incest14 × 6. Victim of sexual abuseZ411. Sexual abuse counselling
**2.** **Physical abuse**How often did a parent or adult in your home ever hit, beat, kick or physically hurt you in any way? This does not include gentle smacking for punishment. Once or more than once	63CB. Risk of non-accidental injury13ZT. At risk of physical abuse14 × 0. History of physical abuse13W40 Child/parent violence14 × 5. Victim of physical abuseZ412. Physical abuse counselling
**3.** **Verbal abuse**How often did a parent or adult in your home ever swear at you, insult you, or put you down? More than once	13ZR. At risk of emotional/psychological abuse14 × 2. History of emotional abuse14 × 7. Victim of emotional abuseZV4H2 [V]Hostility towards and scapegoating of child
**4.** **Domestic violence**How often did your parents or adults in your home ever slap, hit, kick, punch or beat each other up? Once or more than once	13HP6 Violence between parents13VF. At risk violence in the home14 × 3. History of domestic violence14 × 8. Victim of domestic violence14XD. History of domestic abuseZ415. Domestic abuse counselling
**5.** **Parental separation**Were your parents ever separated or divorced? Yes	ZU273 Deserted by fatherZU274 Deserted by mother13W90 Single parent family, mother present13W91 Single parent family, father present
**6.** **Mental illness**Did you live with anyone who was depressed, mentally ill or suicidal? Yes	No codes available
**7.** **Alcohol abuse**Did you live with anyone who was a problem drinker or alcoholic? Yes	63 C7. Maternal alcohol abuse^a^
**8.** **Drug abuse**Did you live with anyone who used illegal street drugs or who abused prescription medications? Yes	12 × 1. Both parents misuse drugs12 × 2. Paternal drug misuse63C6. Maternal drug abuse
**9.** **Incarceration**Did you live with anyone who served time or was sentenced to serve time in a prison or young offender’s institution? Yes	13I7. Imprisonment of family member13Hg. On conditional probation

ACE = adverse childhood experience.

^a^There is no Read code for paternal alcohol abuse.

Read codes that were likely to be direct consequence of ACEs were also categorised and counted. They were based on the codes used when recording child safeguarding concerns or actions;^11^ for example, becoming adopted or entering the care system. **Supplementary Table 1** describes the codes included as ‘childhood consequence of ACEs’.

Based on additional information available from Read codes, categories that described wider childhood adversity, such as ‘inadequate childhood experience’, problems at school, and neglect, were also included. **Supplementary Table 2** describes the Read codes included for these categories.

Codes recorded for adult events relating to ACEs, and those representing potential risk for intergenerational transmission of ACEs,^12^ were also extracted. Read codes were thus categorised into adult experience of abuse: physical, emotional, sexual, domestic, and external violence, general maltreatment, neglect, and evidence of adult involvement in a child safeguarding issue. This was to enable, in this first study about ACEs in the GPR the capture of wider issues relating to adversity experiences. **Supplementary Table 3** describes these Read codes. Careful consideration was given to the meaning of each Read code; for example, ‘history of childhood sexual abuse’ recorded in adulthood signifies an ACE.

The NHS TTP^13^ who produced the GP dataset^14^ produced a frequency count of codes from the dataset at this stage, extracted by ‘event age’ reflecting the date assigned to the Read code in the GPR. This was to ensure events that took place in adulthood were excluded from the ACE questionnaire categories.

### Natural language processing

There was limited literature^15^ allowing estimation of code recording rates and it was predicted that coding of these ACE related codes may be low. Therefore, an additional anonymised method of extracting information was used and tested in partnership with the TTP. This ‘text extract’ used NLP software to identify bigrams or trigrams (pairs or triplets of words) commonly associated with the extracted Read codes in the free text portion of the GPR.^14^ A limitation of this method is that Read codes need to be commonly used to be included in this process; so it can be viewed as enhancing non-Read-coded data from some GPs that other GPs already Read code. Bigrams and trigrams that occurred <10 times in the dataset were discarded.

The remaining bigrams and trigrams were examined by one author for specificity and usefulness. For example:

- *‘and she said’* non-specific and hence useless — discarded
- *‘cause for concern’* probably specific and useful — required a ‘concordance report’
- ‘*child protection’* definitely specific and useful — included and checked with a ‘concordance report’

A concordance report was produced for the retained bigrams/trigrams. This separates out a section of text for each GPR containing the bigram/trigram. Redaction software was used to remove identifiers. The bigrams/trigrams were then reviewed by one author in this anonymised GPR context to make a decision whether or not to include. Inclusion only occurred if all sections of the GPR accurately captured the sense of the ACE domain.

This then led to a final list of bigrams/trigrams allocated a ‘dummy’ Read code which was applied to the data set and the analysis proceeded from there. This analysis does not allow quantification of the error rate because permissions did not allow validation of the results with the original non-anonymised GPR.

### Missed appointment categories

As reported previously, patients were categorised into the following missed appointment categories: zero group (zero missed appointments), low (average of <1 per year), medium (average of ≥1 and ≤2 per year), and high (average of >2 per year). This was calculated from the number of missed GP appointments over a 3-year period.^7,8^

## Results

Records from 824 374 GP patients were available for analysis; 688 725 GPRs were included because they had any Read code associated with a GP appointment.

### Recording of ACEs

The frequencies with which Read codes were recorded is shown in Table 2, along with the results for each ACE questionnaire domain obtained through utilising NLP, then for consequences of ACE for children and wider childhood adversity experiences, and finally for adult consequences of ACEs.

**Table 2.** ACE questionnaire domains mapped onto Read codes; childhood consequences of ACEs; wider childhood adversity; and adult consequences of ACEs; frequency counts of Read codes recorded and the NLP process

**ACE domain**	**Frequency count of Read codes per patient (% sample**)	**Bi/trigram retrieved then sense check edited**	**Bi/trigrams retained after concordance check**	**Conclusion**
**Mapped onto ACE questionnaire**				
Sexual abuse	1306 (0.19)	9/105 bigrams15/119 trigrams retained	1 trigram retained‘sexual abuse by’	Insufficient to proceed with generating dummy Read code
Physical abuse	279 (0.04)	1/17 bigrams4/24 trigrams retained	0 retained	Insufficient to proceed with generating dummy Read code
Verbal abuse	76 (0.01)	Insufficient data	–	NLP not possible
Domestic violence	1017 (0.15)	Insufficient data	–	NLP not possible
Parental separation	16 (0.002)	Insufficient data	–	NLP not possible
Mental illness	No Read Code linked to this domain	–	–	NLP not possible
Alcohol abuse	74 (0.01)	0/5 bigrams0/5 trigrams retained	–	NLP not possible
Drug abuse	83 (0.01)	1/5 bigrams1/4 trigrams retained	1 trigram retained‘on methadone programme’	Insufficient to proceed with generating dummy Read code
Incarceration	25 (0.003)	Insufficient data	–	NLP not possible
**Childhood consequences of ACEs**	11 819 (1.72)	224/998 bigrams412/998 trigrams retained	Data accuracy poor as mixture of ACE exposures and consequences identified	Codes insufficiently specific to the category of consequences of ACE to proceed
**Wider childhood adversity**				
‘Inadequate childhood experience’	231 (0.03)	0/4 bigrams0/7 trigrams retained	–	NLP not possible
Problems at school	621 (0.09)	1/137 bigrams10/83 trigrams retained	1 trigram retained‘by school psychologist’	Insufficient to proceed with dummy Read code
Neglect	17 (0.002)	Insufficient data	–	NLP not possible
**Adult consequences of ACEs**	10 163 (1.48)	86/1466 bigrams219/1468 trigrams retained	Data accuracy poor despite event age method; accuracy of exposure in childhood versus adulthood, and perpetrator versus victim status	Codes insufficiently specific to the category of adult consequences of ACE to proceed

ACE = adverse childhood experience. NLP = natural language processing.

In total, 0.41% of the sample had any Read code recorded that mapped onto the ACE questionnaire domains: 1.72% of the sample had a code recorded that indicated a childhood consequence of ACE, and 0.12% had coding about wider aspects of ACE (such as neglect). A further 1.48% of the sample had a code recorded that indicated an adult consequence of ACEs.

#### ACEs and missed appointments

**Figure 1.**
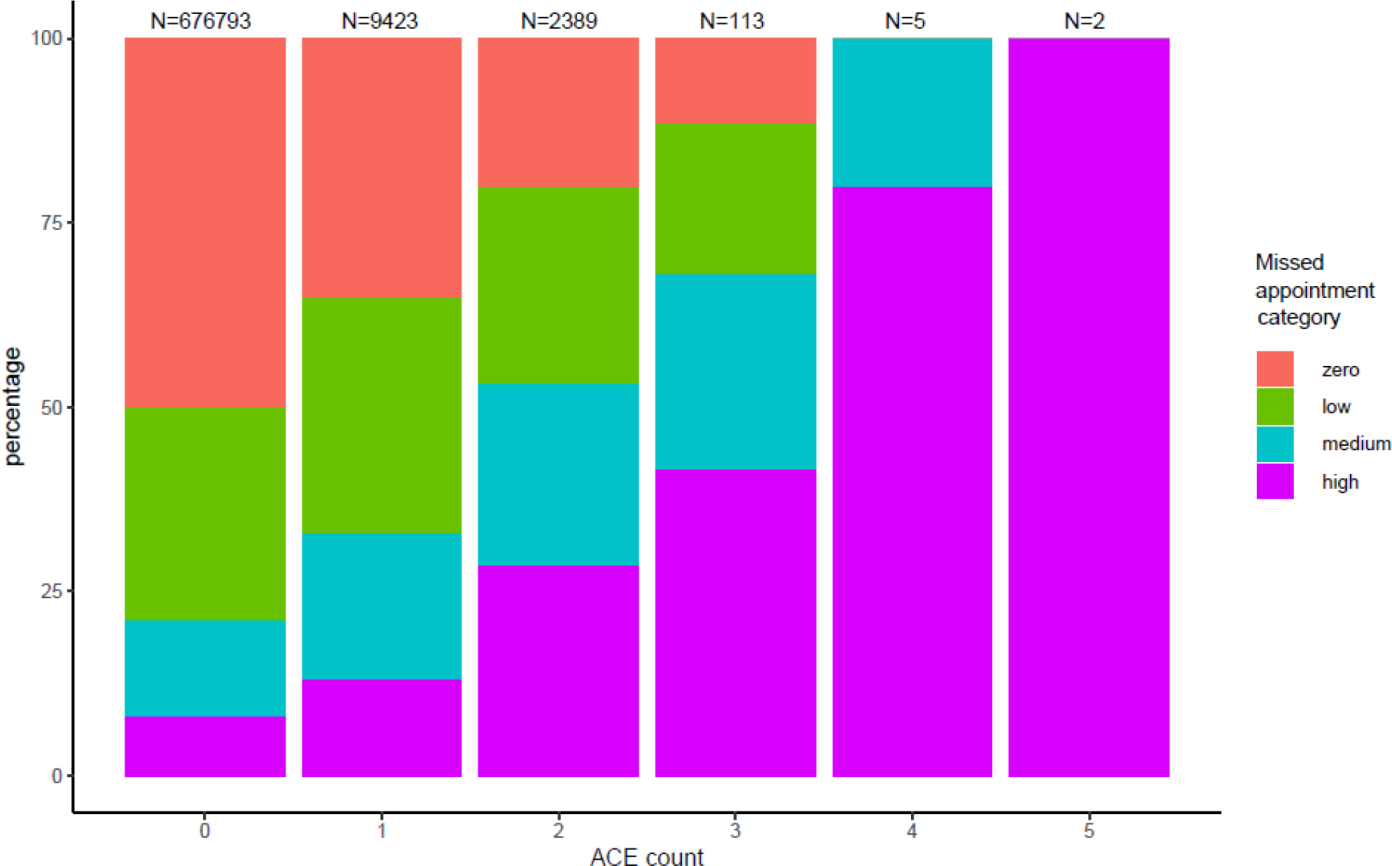
Cumulative ACEs by missed appointment category. ACE = adverse childhood experience.

**Figure 2.**
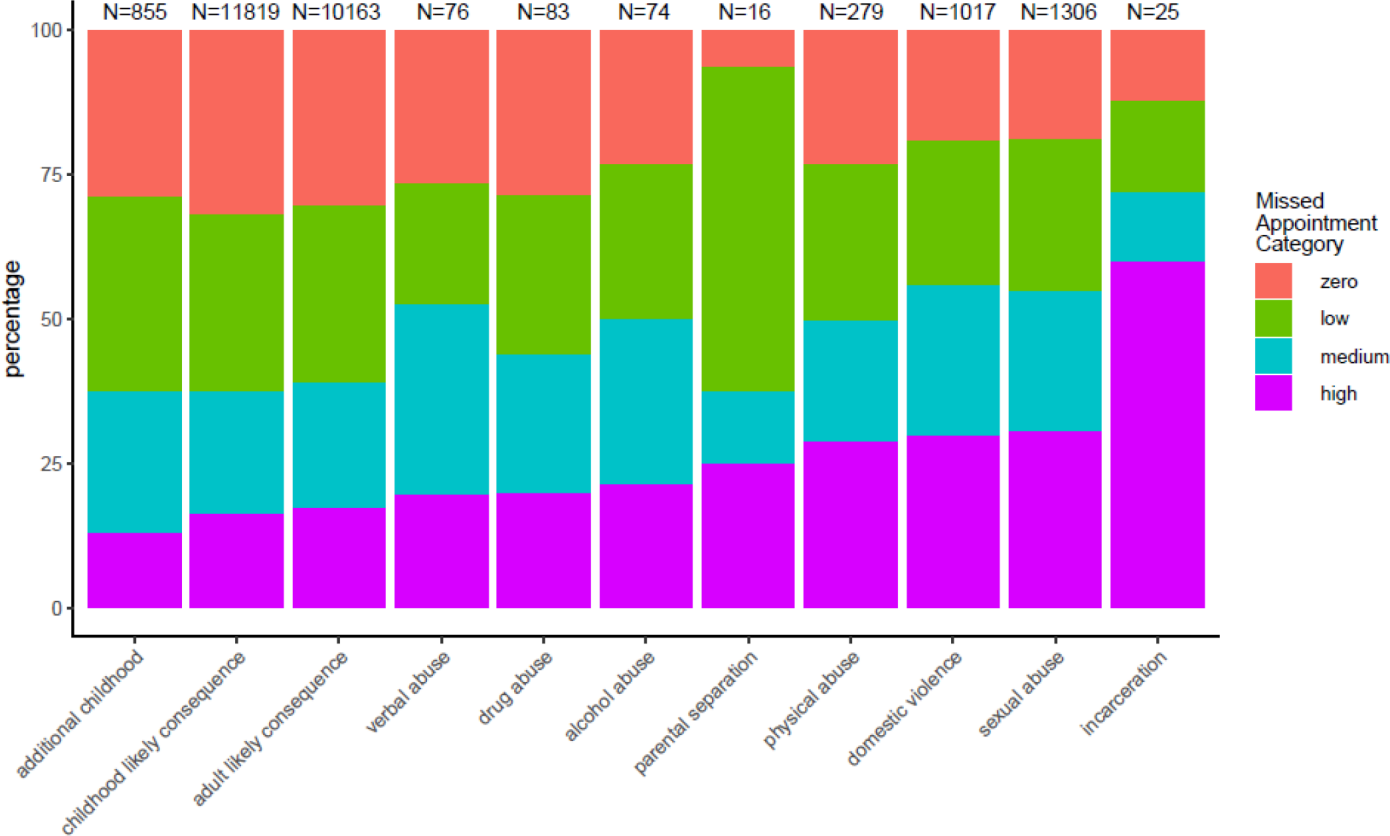
ACE domain by missed appointment category. ACE = adverse childhood experience. **Supplementary Table 4** contains the cross-tabulation tables from which Figure 2 was derived.

Despite the low recording of Read codes that mapped onto ACE domains there was a relationship between having recorded ACEs and missing more GP appointments (Figure 1 and Figure 2).

This data shows a substantially increased risk of missing appointments among people with recorded ACEs.

## Discussion

### Summary

This large retrospective cohort study using GPRs provides striking evidence that ACEs are rarely coded in NHS general practice. This applied to coding associated with standard questionnaires commonly used in adult population surveys, and codes relating to wider experience of adversity related to inadequate childhood experiences, problems at school, and neglect.

The codes relating to childhood consequences of ACEs which correspond broadly to child safeguarding concerns are more prevalent at 1.72%. Recorded adult consequences were 1.48%.

Despite low recording rates there was an association between the current recording of ACEs and a higher risk of missing GP appointments. This supported the hypothesis that the experience of adversity in childhood is likely to be associated with patterns of high levels of missed GP appointments. Serial missed appointments may be a measurable proxy for low engagement in care,^7^ which can in turn be one of a range of health harming behaviours linked to experiencing adversity in childhood.^3,4,16,17^

### Strengths and limitations

One strength of this study is that comprehensive GPRs from a representative sample of general practices were used, giving coverage of one-sixth of the Scottish population.

Efforts were also made to augment the low rate of recording of ACE codes using natural language processing,^14^ but this added little to the coded prevalence, largely due to the complex textual context in which the adversity was recorded. Sometimes the adversity mentioned was that of a family member (for example, the patient’s child), on other occasions it was recording that no adversity was reported. Occasionally the text was describing a perpetrator. Therefore, the decision-making process that underlies a member of the GP team’s coding of adversity or adversity outcome is incompletely understood. However, one potential limitation in interpretation of the findings is ascertainment bias: coding may be more likely if the patient presents with difficulties or is causing concern to a clinician, such as repeatedly failing to attend appointments. Moreover, published guidance about which Read codes to include in the analysis was sparse and decisions about what to include were made by only one GP with moderation by one further GP. For future work investigating the coding and recording of complex issues in the GPR, more robust validation of included codes for analysis and more sophisticated language rules would need to be tested, in addition to event age, and the context in which clinicians decide to code adversity or its consequences should be explored. Finally, childhood adverse events (for example, marital separation or parental drug use) may be reflected in the records of family members rather than in those of the index child so this should be factored into study design.

### Comparison with existing literature

There has been no prior published work examining recording of ACEs in the GPR. Comparison of the current results with recent population studies from England and Wales reporting ACEs suggest there is significant under-recording of ACEs in the GPR. Population representative samples of adults in England and Wales record prevalence of ACEs to be much higher than found in this study of coding in GPRs. For example, in the English study the rates of reported verbal abuse were 24.3%, physical abuse 18.2%, and sexual abuse 14.8%,^3^ comparable to the Welsh study,^4^ compared with 0.01%, 0.04%, and 0.19%, respectively, coded in the current sample. A total of 0.41% of patients had a record of any code that mapped onto the ACE questionnaire, contrasting with 47% in population samples.^4,18^ Recent evidence from an analysis of ACEs in the Scottish ‘Growing up In Scotland’ birth cohort at age 8^19^ confirms that Scottish data would be expected to be similar to England and Wales.

The codes relating to childhood consequences of ACEs, which correspond broadly to child safeguarding concerns, had a prevalence of 1.72%, compared to 0.9% recorded in a broadly similar sample from 2010 in England that investigated GP coding of childhood maltreatment.^15^ Recorded adult consequences were 1.48%, and there are no previous studies the authors are aware of that investigate adult outcomes of ACEs. Proportions were never the less increased only modestly by including these data.

This study has established for the first time an association between missed appointments and ACE-related coding.

### Implications for research and practice

This study confirms that the current state of coded data in relation to ACEs is poor. The Read code system is not fit for purpose if recording of historical or current adversity were to become part of routine data recording. Future planning of replacement coding systems should take this into account.

There exist ongoing tensions about what GPs and other practice team members record and code in the GPR. In part, this is likely to be due to constraints on time available for recording and coding, and tensions in the consultation between focus on the screen for recording, and attention to the patient. There may also be concerns about recording detailed sensitive information in a GPR both about the patient and a third party such as a family member. It is suspected that much of this information is instead held in professionals’ memories, though a lack of relational continuity of care may diminish the value of this approach. Another factor in low levels of recording may be that there is no current external incentive to record and code adversity, unlike the situation with active child safeguarding concerns; availability of templates is sparse and there is no external accountability or payment in the UK. As debate about ACE-informed practice develops, this evidence is an important reminder of the significant further research and support that would be required for general practices to incorporate recording ACEs into their everyday clinical practice as a significant risk factor for increased morbidity and premature mortality.

Despite data recording being poor, there is evidence that patients who miss more than two GP appointments per year are more likely to have had an ACE. This adds a further dimension to existing evidence that patients who serially miss appointments are more likely to be socially vulnerable and have poorer health outcomes.
